# Use of Tobacco Products, Alcohol, and Other Substances Among High School Students During the COVID-19 Pandemic — Adolescent Behaviors and Experiences Survey, United States, January–June 2021

**DOI:** 10.15585/mmwr.su7103a2

**Published:** 2022-04-01

**Authors:** Nancy D. Brener, Michele K. Bohm, Christopher M. Jones, Samantha Puvanesarajah, Leah Robin, Nicolas Suarez, Xiaoyi Deng, R. Lee Harding, Davia Moyse

**Affiliations:** ^1^Division of Adolescent and School Health, National Center for HIV, Viral Hepatitis, STD, and TB Prevention, CDC; ^2^Divsion of Population Health, National Center for Chronic Disease Prevention and Health Promotion, CDC; ^3^Office of the Director, National Center for Injury Prevention and Control, CDC; ^4^Office on Smoking and Health, National Center for Chronic Disease Prevention and Health Promotion, CDC; ^5^ICF International, Rockville, Maryland

## Abstract

The COVID-19 pandemic has been associated with established risk factors for adolescent substance use, including social isolation, boredom, grief, trauma, and stress. However, little is known about adolescent substance use patterns during the pandemic. CDC analyzed data from the Adolescent Behaviors and Experiences Survey, an online survey of a probability-based, nationally representative sample of public- and private-school students in grades 9–12 (N = 7,705), to examine the prevalence of current use of tobacco products, alcohol, and other substances among U.S. high school students. Prevalence was examined by demographic characteristics and instructional models of the students’ schools (in-person, virtual, or hybrid). During January–June 2021, 31.6% of high school students reported current use of any tobacco product, alcohol, or marijuana or current misuse of prescription opioids. Current alcohol use (19.5%), electronic vapor product (EVP) use (15.4%), and marijuana use (12.8%) were more prevalent than prescription opioid misuse (4.3%), current cigarette smoking (3.3%), cigar smoking (2.3%), and smokeless tobacco use (1.9%). Approximately one third of students who used EVPs did so daily, and 22.4% of students who drank alcohol did so ≥6 times per month. Approximately one in three students who ever used alcohol or other drugs reported using these substances more during the pandemic. The prevalence of substance use was typically higher among non-Hispanic American Indian or Alaska Native students, older students, and gay, lesbian, or bisexual students than among students of other racial or ethnic groups, younger students, and heterosexual students. The prevalence of alcohol use also was higher among non-Hispanic White students than those of other racial or ethnic groups. Students only attending school virtually had a lower prevalence of using most of the substances examined than did students attending schools with in-person or hybrid models. These findings characterizing youth substance use during the pandemic can help inform public health interventions and messaging to address these health risks during and after the COVID-19 pandemic.

## Introduction

During the COVID-19 pandemic, many adolescents experienced factors that might increase risk for substance use, including social isolation and boredom; stress from fear about COVID-19; grief; economic, housing, and food insecurity; and disruption to medical, mental health, and social services (*1*). However, little is known about adolescent substance use patterns during the pandemic. Studies examining changes in substance use among various populations of adolescents during the early stages of the pandemic (2020) have shown mixed results. Although certain studies demonstrated declines in the use of electronic vapor products (EVPs) and binge drinking (*2*–*4**,**5*), one study found increases in the frequency of alcohol and marijuana use (*5*), and others indicated no change in alcohol use (*3*), binge drinking, or marijuana use (*4*). Inconsistent results across studies might be explained by differences in populations, time frames, and data collection methods. Of note, none of these studies used probability samples of U.S. high school students in all grades.

Additional factors might contribute to changes in adolescent substance use. For example, because youths obtain most EVPs from social sources (*6*), access to these products during the pandemic likely decreased. However, more permissive alcohol policies during the pandemic (e.g., home delivery) might have increased alcohol availability and weakened established age-gating barriers for youths attempting to purchase alcohol. In addition, parental behaviors related to pandemic stressors might have influenced youth substance use. A longitudinal study found that 16% of parents newly allowed their adolescent children to drink alcohol with the family during the pandemic (*7*).

To better understand adolescent substance use during the COVID-19 pandemic, this report presents data from a national survey conducted during January–June 2021 to examine use of tobacco products, alcohol, and marijuana and the misuse of prescription opioids among U.S. high school students during the 30 days before the survey. Findings in this report can help inform prevention strategies and public health messaging during and after the pandemic.

## Methods

### Data Source

Data from the Adolescent Behaviors and Experiences Survey (ABES) conducted by CDC during January–June 2021 were used to assess student behaviors and experiences during the COVID-19 pandemic. ABES was a one-time, probability-based online survey of U.S. high school students. ABES used a stratified, three-stage cluster sampling approach to obtain a nationally representative sample of public- and private-school students in grades 9–12 in the 50 U.S. states and the District of Columbia (N = 7,705). Participation in ABES was voluntary; each school and teacher decided whether students completed the survey during instructional time or on their own time. Additional information about ABES sampling, data collection, response rates, and processing is available in the overview report of this supplement (*8*). The ABES questionnaire, data sets, and documentation are available at https://www.cdc.gov/healthyyouth/data/abes.htm.

### Measures

The prevalence of current use (≥1 day during the 30 days before the survey) of six substances (EVPs, cigarettes, cigars, smokeless tobacco, alcohol, and marijuana), plus current misuse of prescription opioids, was examined (Table 1). Also examined were the prevalence and frequency of current alcohol use and binge drinking, the largest number of alcoholic drinks consumed in a row, frequency of EVP use, source of EVPs, source of alcohol, and changes in alcohol and drug use during the COVID-19 pandemic (Table 1). Two composite measures were created: 1) any current tobacco product use, defined as any use of EVPs, cigarettes, cigars, or smokeless tobacco during the 30 days before the survey, and 2) current use of any tobacco product, alcohol, or marijuana or prescription opioid misuse.

**TABLE 1:** Variables, questions, and analytic coding for tobacco product, alcohol, and other substance use — Adolescent Behaviors and Experiences Survey, United States, January–June 2021

Variable	Question	Analytic coding
Current electronic vapor product use	During the past 30 days, on how many days did you use an electronic vapor product?*	≥1 day versus 0 days
Frequent current electronic vapor product use	During the past 30 days, on how many days did you use an electronic vapor product?*	≥20 days versus 1 or 2 days, 3–5 days, 6–9 days, or 10–19 days
Current cigarette smoking	During the past 30 days, on how many days did you smoke cigarettes?	≥1 day versus 0 days
Current cigar smoking	During the past 30 days, on how many days did you smoke cigars, cigarillos, or little cigars?	≥1 day versus 0 days
Current smokeless tobacco use	During the past 30 days, on how many days did you use chewing tobacco, snuff, dip, snus, or dissolvable tobacco products, such as Copenhagen, Grizzly, Skoal, or Camel Snus? (Do not count any electronic vapor products.)	≥1 day versus 0 days
Current alcohol use	During the past 30 days, on how many days did you have at least one drink of alcohol?^†^	≥1 day versus 0 days
Frequent current alcohol use	During the past 30 days, on how many days did you have at least one drink of alcohol?	≥6 days versus 1 day, 2 days, or 3–5 days
Current binge drinking	During the past 30 days, on how many days did you have four or more drinks of alcohol in a row, that is, within a couple of hours (if you are female) or five or more drinks of alcohol in a row, that is, within a couple of hours (if you are male)?	≥1 day versus 0 days
Frequent current binge drinking	During the past 30 days, on how many days did you have four or more drinks of alcohol in a row, that is, within a couple of hours (if you are female) or five or more drinks of alcohol in a row, that is, within a couple of hours (if you are male)?	≥6 days versus 1 day, 2 days, or 3–5 days
Largest number of drinks in a row	During the past 30 days, what is the largest number of alcoholic drinks you had in a row, that is, within a couple of hours?	Females: ≥8 drinks versus 4 drinks, 5 drinks, or 6 or 7 drinks Males: ≥8 drinks versus 5 drinks or 6 or 7 drinks
Current marijuana use	During the past 30 days, how many times did you use marijuana?^§^	≥1 time versus 0 times
Current prescription opioid misuse	During the past 30 days, how many times have you taken prescription pain medicine without a doctor’s prescription or differently than how a doctor told you to use it?^¶^	≥1 time versus 0 times
Source of electronic vapor products	During the past 30 days, how did you usually get your electronic vapor products? (Select only one response.)	NA
Source of alcohol	During the past 30 days, how did you usually get the alcohol you drank?	NA
Drank more alcohol during the COVID-19 pandemic	Do you agree or disagree that you drank more alcohol during the COVID-19 pandemic than before it started?	Strongly agree or agree versus not sure, disagree, or strongly disagree
Used more drugs during the COVID-19 pandemic	Do you agree or disagree that you used drugs more during the COVID-19 pandemic than before it started? (Count using marijuana, synthetic marijuana, cocaine, prescription pain medicine without a doctor’s prescription, and other illegal drugs.)	Strongly agree or agree versus not sure, disagree, or strongly disagree

**Abbreviation:** NA = not applicable.

* Electronic vapor products were defined in a preamble that read, “The next three questions ask about electronic vapor products, such as Juul, Smok, Suorin, Vuse, and blu. Electronic vapor products include e-cigarettes, vapes, vape pens, e-cigars, e-hookahs, hookah pens, and mods.”

^†^ Alcohol was defined in a preamble that read, “The next five questions ask about drinking alcohol. This includes drinking beer, wine, flavored alcoholic beverages, and liquor such as rum, gin, vodka, or whiskey. For these questions, drinking alcohol does not include drinking a few sips of wine for religious purposes.”

^§^ Marijuana was defined in a preamble that read, “The next three questions ask about marijuana use. Marijuana also is called pot or weed. For these questions, do not count CBD-only or hemp products, which come from the same plant as marijuana, but do not cause a high when used alone.”

^¶^ Prescription opioid misuse was defined in a preamble that read, “The next two questions ask about the use of prescription pain medicine without a doctor’s prescription or differently than how a doctor told you to use it. For these questions, count drugs such as codeine, Vicodin, OxyContin, hydrocodone, and Percocet.”

Assessed demographic characteristics included sex, race and ethnicity (non-Hispanic American Indian or Alaska Native [AI/AN], non-Hispanic Asian [Asian], non-Hispanic Black [Black], Hispanic or Latino of any race [Hispanic], non-Hispanic persons of multiple races [multiracial], non-Hispanic Native Hawaiian or other Pacific Islander [NH/OPI], or non-Hispanic White [White]), grade (9, 10, 11, or 12), and sexual identity (heterosexual; gay, lesbian, or bisexual; or other or questioning). Analyses also examined the instructional model of the school the student was attending (virtual, in-person, or hybrid).

### Analysis

Weighted prevalence estimates and corresponding 95% CIs were calculated, and *t*-tests were used to assess differences between groups. Differences between prevalence estimates were considered statistically significant if the *t*-test p value was <0.05. Only statistically significant differences in prevalence estimates are reported. Analyses were completed using SUDAAN (version 11.0.1; RTI International) to account for the complex survey design and weighting.

## Results

During January–June 2021, 31.6% of high school students reported current use of any tobacco product, alcohol, or marijuana or current misuse of prescription opioids. Current alcohol use (19.5%), EVP use (15.4%), and marijuana use (12.8%) were more prevalent among high school students than prescription opioid misuse (4.3%), current cigarette smoking (3.3%), cigar smoking (2.3%), and smokeless tobacco use (1.9%). For tobacco product use, differences by demographic characteristics varied by type of tobacco product (Table 2); use of these products was most prevalent among AI/AN, White, and multiracial students and least prevalent among Asian students. Tobacco product use was more prevalent among 12th-grade students than students in lower grades; more prevalent among gay, lesbian, or bisexual students than heterosexual or other or questioning students; and least prevalent among students attending virtual-only schools. Among students who currently used EVPs, 38.0% used them on at least 20 of the 30 days before the survey and 30.9% used them on all 30 days. Students who currently used EVPs most commonly obtained them by getting or buying them from a friend, family member, or someone else (52.4%).

**TABLE 2:** Percentage of high school students who currently used tobacco products,* by selected characteristics and type of tobacco product — Adolescent Behaviors and Experiences Survey, United States, January–June 2021

Characteristic	Current electronic vapor product use^†^	Current cigarette use^§^	Current cigar use^¶^	Current smokeless tobacco use**	Any current tobacco product use^††,§§^
	% (95% CI)	% (95% CI)	% (95% CI)	% (95% CI)	% (95% CI)
**Sex**					
Female	16.8 (13.4–20.8)	3.0 (2.1–4.5)	1.3 (1.0–1.9)	0.6 (0.4–1.0)	16.9 (13.7–20.7)
Male	13.9 (11.9–16.3)	3.6 (2.6–4.9)	3.1 (2.4–4.1)	3.2 (2.2–4.8)	14.3 (12.3–16.6)
**Race and ethnicity**					
American Indian or Alaska Native, non-Hispanic	23.5 (12.3–40.1)	8.9 (5.6–13.8)	4.1 (2.2–7.7)	7.1 (3.3–14.8)	24.0 (11.5–43.5)
Asian, non-Hispanic	4.4 (2.3–8.2)	0.3 (0.1–1.3)	0.5 (0.1–1.8)	1.3 (0.3–5.2)	4.4 (2.3–8.4)
Black, non-Hispanic	10.6 (8.5–13.2)	0.9 (0.3–2.3)	2.6 (1.7–4.1)	0.4 (0.2–0.9)	11.0 (8.7–13.8)
Hispanic or Latino (all races)	9.7 (7.2–12.9)	1.8 (1.2–2.8)	1.3 (0.9–2.1)	0.8 (0.5–1.5)	9.4 (7.1–12.3)
Multiracial, non-Hispanic	17.6 (13.2–23.0)	4.1 (2.0–8.3)	5.4 (3.2–9.0)	1.1 (0.5–2.5)	19.4 (14.5–25.5)
Native Hawaiian or other Pacific Islander, non-Hispanic	—^¶¶^	1.2 (0.1–11.0)	3.2 (0.4–21.4)	3.2 (0.4–21.4)	—
White, non-Hispanic	20.3 (17.2–23.9)	4.9 (3.6–6.5)	2.4 (1.7–3.4)	3.0 (2.0–4.3)	20.7 (17.6–24.0)
**Grade**					
9	13.3 (10.0–17.4)	2.7 (1.6–4.3)	1.9 (1.2–3.0)	1.2 (0.7–2.2)	13.0 (9.9–16.9)
10	12.3 (9.7–15.6)	2.5 (1.6–3.9)	1.9 (1.3–2.8)	1.5 (0.8–3.0)	12.6 (10.0–15.6)
11	16.1 (13.1–19.7)	3.6 (2.5–5.0)	1.6 (1.0–2.4)	1.8 (1.1–2.8)	16.8 (13.8–20.3)
12	20.4 (17.3–23.8)	4.5 (2.6–7.6)	3.6 (2.4–5.4)	3.0 (1.8–5.1)	20.8 (17.4–24.6)
**Sexual identity**					
Heterosexual	14.7 (12.1–17.6)	2.9 (2.1–3.9)	2.0 (1.6–2.7)	2.0 (1.4–3.0)	14.9 (12.5–17.7)
Gay, lesbian, or bisexual	20.9 (16.8–25.7)	6.2 (3.6–10.3)	3.5 (2.3–5.3)	1.5 (0.8–2.7)	21.6 (17.3–26.5)
Other or questioning	16.1 (12.2–21.1)	3.3 (1.9–5.6)	1.4 (0.7–2.8)	0.7 (0.3–2.2)	15.8 (12.0–20.5)
**Instructional model of school**					
In-person only	25.2 (13.9–41.2)	5.2 (2.5–10.8)	3.4 (1.8–6.5)	2.9 (1.7–5.1)	25.5 (13.9–42.0)
Virtual only	9.1 (7.3–11.2)	1.4 (0.9–2.0)	1.0 (0.6–1.8)	0.5 (0.2–1.2)	9.0 (7.1–11.4)
Hybrid	17.2 (14.4–20.5)	3.9 (2.8–5.3)	2.6 (2.0–3.4)	2.4 (1.6–3.4)	17.5 (14.8–20.7)
**Total**	**15.4 (13.0–18.1)**	**3.3 (2.5–4.4)**	**2.3 (1.8–2.9)**	**1.9 (1.3–2.8)**	**15.6 (13.3–18.2)**

* Weighted percentages. See Table 1 for variable definitions.

^†^ Pairwise *t*-tests indicate significant differences (p<0.05) between the following subgroups of students: non-Hispanic American Indian or Alaska Native versus non-Hispanic Asian; non-Hispanic Asian versus non-Hispanic Black, Hispanic, non-Hispanic multiracial, and non-Hispanic White; non-Hispanic Black versus non-Hispanic White and non-Hispanic multiracial; Hispanic versus non-Hispanic multiracial and non-Hispanic White; grade 9 versus grade 12; grade 10 versus grade 12; grade 11 versus grade 12; heterosexual versus gay, lesbian, and bisexual; in-person versus virtual; and virtual versus hybrid.

^§^ Pairwise *t*-tests indicate significant differences (p<0.05) between the following subgroups of students: non-Hispanic American Indian or Alaska Native versus non-Hispanic Asian, non-Hispanic Black, Hispanic, non-Hispanic Native Hawaiian or other Pacific Islander, and non-Hispanic White; non-Hispanic Asian versus Hispanic, non-Hispanic multiracial, and non-Hispanic White; non-Hispanic Black versus non-Hispanic multiracial and non-Hispanic White; Hispanic versus non-Hispanic White; non-Hispanic Native Hawaiian or other Pacific Islander versus non-Hispanic White; heterosexual versus gay, lesbian, or bisexual; and virtual versus hybrid.

^¶^ Pairwise *t*-tests indicate significant differences (p<0.05) between the following subgroups of students: female versus male; non-Hispanic American Indian or Alaska Native versus non-Hispanic Asian; non-Hispanic Asian versus non-Hispanic Black, Hispanic, non-Hispanic multiracial, and non-Hispanic White; non-Hispanic Black versus Hispanic; Hispanic versus non-Hispanic multiracial and non-Hispanic White; heterosexual versus gay, lesbian, or bisexual; gay, lesbian, or bisexual versus other or questioning; in-person versus virtual; and virtual versus hybrid.

** Pairwise *t*-tests indicate significant differences (p<0.05) between the following subgroups of students: female versus male; grade 9 versus grade 12; non-Hispanic American Indian or Alaska Native versus non-Hispanic Black, Hispanic, and non-Hispanic multiracial; non-Hispanic Black versus non-Hispanic White; Hispanic versus non-Hispanic White; non-Hispanic multiracial versus non-Hispanic White; heterosexual versus other or questioning; in-person versus virtual; and virtual versus hybrid.

^††^ Smoked cigarettes or cigars or used smokeless tobacco or an electronic vapor product. To be consistent with other CDC surveillance systems, variable calculated among students who answered all four questions related to tobacco product use.

^§§^ Pairwise *t*-tests indicate significant differences (p<0.05) between the following subgroups of students: grade 9 versus grade 12; grade 10 versus grades 11 and 12; non-Hispanic American Indian or Alaska Native versus non-Hispanic Asian; non-Hispanic Asian versus non-Hispanic Black, Hispanic, non-Hispanic multiracial, and non-Hispanic White; non-Hispanic Black versus non-Hispanic multiracial and non-Hispanic White; Hispanic versus non-Hispanic multiracial and non-Hispanic White; heterosexual versus gay, lesbian, or bisexual; gay, lesbian, or bisexual versus other or questioning; in-person versus virtual; and virtual versus hybrid.

^¶¶^ Dashes indicate that results are suppressed because n<30.

Both current alcohol use and binge drinking varied by demographic characteristics (Table 3). These behaviors were more prevalent among female than male students, most prevalent among White and multiracial students, and more prevalent among students in higher than lower grades; among gay, lesbian, or bisexual students than heterosexual students; and among students attending hybrid schools than those attending virtual-only schools. Among the 43.1% of students who had ever drunk alcohol, 29.6% strongly agreed or agreed that they drank more alcohol during the COVID-19 pandemic. Drinking more alcohol during the pandemic varied by race and ethnicity but not by grade, sexual identity, or instructional model. Among students who currently drank alcohol, 22.4% drank on ≥6 of the 30 days before the survey. Among the 7.7% of students who reported current binge drinking, 21.2% binge drank on ≥6 of the 30 days before the survey and 39.2% consumed eight or more drinks in a row. Students who currently drank alcohol most commonly obtained it by someone giving it to them (38.3%).

**TABLE 3:** Percentage of high school students who currently drank alcohol or were binge drinking and percentage who strongly agreed or agreed that they drank more alcohol during the COVID-19 pandemic than before it started,* by selected characteristics — Adolescent Behaviors and Experiences Survey, United States, January–June 2021

Characteristic	Current alcohol use^†^	Current binge drinking^§^	Drank more alcohol during the COVID-19 pandemic^¶,^**
	% (95% CI)	% (95% CI)	% (95% CI)
**Sex**			
Female	22.4 (18.5–27.0)	9.5 (6.6–13.5)	27.8 (24.6–31.3)
Male	16.4 (14.3–18.7)	5.9 (4.6–7.6)	31.9 (28.1–36.0)
**Race and ethnicity**			
American Indian or Alaska Native, non-Hispanic	20.5 (12.7–31.5)	5.8 (3.0–11.0)	14.5 (6.7–28.7)
Asian, non-Hispanic	14.9 (10.0–21.8)	1.8 (0.9–3.6)	30.3 (22.0–40.1)
Black, non-Hispanic	11.0 (8.6–13.9)	2.4 (1.3–4.3)	17.5 (12.5–23.9)
Hispanic or Latino (all races)	16.5 (12.9–20.8)	5.0 (3.4–7.2)	23.6 (19.1–28.7)
Multiracial, non-Hispanic	22.1 (16.7–28.6)	8.5 (6.0–11.7)	34.0 (25.9–43.2)
Native Hawaiian or other Pacific Islander, non-Hispanic	—^††^	—	—
White, non-Hispanic	23.5 (19.4–28.3)	11.2 (8.1–15.1)	33.6 (30.1–37.4)
**Grade**			
9	12.9 (9.9–16.6)	4.6 (2.5–8.2)	28.6 (22.6–35.6)
10	17.1 (14.0–20.8)	5.3 (3.5–7.8)	29.6 (25.0–34.7)
11	21.7 (18.2–25.7)	9.6 (7.5–12.2)	28.4 (23.6–33.8)
12	27.2 (23.8–30.9)	12.1 (9.5–15.2)	31.3 (26.3–36.7)
**Sexual identity**			
Heterosexual	18.8 (15.6–22.5)	7.8 (5.6–10.8)	29.8 (26.4–33.4)
Gay, lesbian, or bisexual	26.4 (22.1–31.3)	9.0 (6.5–12.5)	31.6 (25.7–38.3)
Other or questioning	20.1 (15.1–26.4)	7.5 (4.8–11.7)	24.5 (18.7–31.6)
**Instructional model of school**			
In-person only	24.2 (12.9–40.8)	10.1 (5.0–19.1)	28.9 (14.3–49.8)
Virtual only	13.5 (10.7–16.8)	3.8 (2.5–5.9)	26.3 (22.0–31.2)
Hybrid	21.3 (18.3–24.7)	9.0 (6.7–11.8)	30.5 (27.3–33.9)
**Total**	**19.5 (16.8–22.4)**	**7.7 (5.9–10.1)**	**29.6 (26.9–32.5)**

* Weighted percentages. See Table 1 for variable definitions.

^†^ Pairwise *t*-tests indicate significant differences (p<0.05) between the following subgroups of students: female versus male; non-Hispanic American Indian or Alaska Native versus non-Hispanic Black; non-Hispanic Asian versus non-Hispanic White; non-Hispanic Black versus non-Hispanic White, Hispanic, and non-Hispanic multiracial; non-Hispanic White versus Hispanic; grade 9 versus grades 10, 11, and 12; grade 10 versus grades 11 and 12 grades; grade 11 versus grade 12; heterosexual versus gay, lesbian, or bisexual; and hybrid versus virtual.

^§^ Pairwise *t*-tests indicate significant differences (p<0.05) between the following subgroups of students: female versus male; non-Hispanic American Indian or Alaska Native versus non-Hispanic White; non-Hispanic Asian versus non-Hispanic White, Hispanic, and non-Hispanic multiracial; non-Hispanic Black versus Hispanic, non-Hispanic White, and non-Hispanic multiracial; Hispanic versus non-Hispanic multiracial and non-Hispanic White; grade 9 versus grades 11 and 12; grade 10 versus grades 11 and 12 grades; and hybrid versus virtual.

^¶^ Among students who had ever drunk alcohol.

** Pairwise *t*-tests indicate significant differences (p<0.05) between the following subgroups of students: non-Hispanic American Indian or Alaska Native versus non-Hispanic Asian, non-Hispanic multiracial, and non-Hispanic White; non-Hispanic Asian versus non-Hispanic Black; non-Hispanic Black versus non-Hispanic multiracial and non-Hispanic White; Hispanic versus non-Hispanic multiracial; and non-Hispanic White versus Hispanic.

^††^ Dashes indicate that results are suppressed because n<30.

Nationally, 12.8% of students currently used marijuana and 4.3% currently misused prescription opioids (Table 4). Differences by demographic group varied by substance. Although marijuana use did not differ by sex, prescription opioid misuse was more prevalent among female than male students. Marijuana use was most prevalent among AI/AN students and multiracial students and least prevalent among Asian students, whereas prescription opioid misuse did not vary by race or ethnicity. Both marijuana use and prescription opioid misuse were more prevalent among gay, lesbian, or bisexual students than among heterosexual students; marijuana use also was more prevalent among gay, lesbian, or bisexual students than other or questioning students. Both types of substance use were more prevalent among students attending hybrid schools than those attending virtual-only schools. In addition, prescription opioid misuse was most prevalent among students attending only in-person schools. Among the 33.7% of students who ever used an illicit drug (marijuana, synthetic marijuana, cocaine, or other illegal drug use or prescription opioid misuse), 31.4% strongly agreed or agreed that they used more drugs during the COVID-19 pandemic. Reporting more drug use during the pandemic was least prevalent among Asian students and Hispanic students, more prevalent among 12th-grade students than 9th-grade students, and more prevalent among students attending hybrid schools than those attending virtual-only schools.

**TABLE 4:** Percentage of high school students who currently used marijuana or misused prescription opioids and percentage who strongly agreed or agreed that they used more drugs during the COVID-19 pandemic than before it started,* by selected characteristics — Adolescent Behaviors and Experiences Survey, United States, January–June 2021

Characteristic	Current marijuana use^†^	Current prescription opioid misuse^§^	Used more drugs during the COVID-19 pandemic^¶,^**
	% (95% CI)	% (95% CI)	% (95% CI)
**Sex**			
Female	12.7 (10.2–15.6)	5.4 (4.4–6.5)	30.1 (25.8–34.7)
Male	12.9 (11.2–14.8)	3.2 (2.3–4.4)	33.1 (29.7–36.7)
**Race and ethnicity**			
American Indian or Alaska Native, non-Hispanic	25.9 (15.7–39.7)	6.2 (3.3–11.2)	25.2 (11.1–47.6)
Asian, non-Hispanic	4.2 (2.1–8.2)	3.4 (2.2–5.2)	17.9 (11.0–27.8)
Black, non-Hispanic	13.6 (10.5–17.5)	4.1 (2.9–5.8)	31.6 (24.8–39.3)
Hispanic or Latino (all races)	9.9 (7.3–13.4)	4.6 (3.6–6.0)	22.2 (17.9–27.2)
Multiracial, non-Hispanic	19.8 (14.7–26.2)	4.2 (2.6–6.9)	36.5 (29.0–44.8)
Native Hawaiian or other Pacific Islander, non-Hispanic	—^††^	—	—
White, non-Hispanic	14.0 (11.8–16.5)	4.3 (3.2–5.7)	36.6 (31.8–41.7)
**Grade**			
9	9.4 (7.0–12.7)	5.3 (4.2–6.7)	27.7 (22.2–33.9)
10	10.5 (8.3–13.2)	4.3 (3.2–5.7)	29.7 (23.5–36.8)
11	13.4 (11.3–15.9)	4.0 (2.8–5.7)	30.6 (26.1–35.5)
12	18.4 (15.8–21.4)	3.6 (2.6–4.9)	36.5 (31.4–41.9)
**Sexual identity**			
Heterosexual	12.0 (10.3–13.8)	3.8 (3.0–4.7)	31.1 (27.4–35.0)
Gay, lesbian, or bisexual	18.5 (14.7–22.9)	6.9 (5.2–9.0)	35.7 (28.9–43.0)
Other or questioning	12.1 (9.1–15.8)	5.5 (3.8–7.8)	26.6 (19.0–35.9)
**Instructional model of school**			
In-person only	16.8 (8.4–30.8)	7.3 (5.4–9.6)	30.5 (21.3–41.4)
Virtual only	9.0 (7.0–11.5)	2.8 (1.9–4.1)	24.5 (18.6–31.6)
Hybrid	13.9 (11.9–16.2)	4.7 (3.8–5.9)	33.4 (30.0–37.0)
**Total**	**12.8 (11.1–14.7)**	**4.3 (3.6–5.2)**	**31.4 (28.3–34.7)**

* Weighted percentages. See Table 1 for variable definitions.

^†^ Pairwise *t*-tests indicate significant differences (p<0.05) between the following subgroups of students: non-Hispanic American Indian or Alaska Native versus non-Hispanic Asian and Hispanic; non-Hispanic Asian versus non-Hispanic Black, Hispanic, non-Hispanic multiracial, and non-Hispanic White; Hispanic versus non-Hispanic multiracial and non-Hispanic White; grade 9 versus grades 11 and 12; grade 10 versus grade 12; grade 11 versus grade 12; heterosexual versus gay, lesbian, or bisexual; gay, lesbian, or bisexual versus other or questioning; and virtual versus hybrid.

^§^ Pairwise *t*-tests indicate significant differences (p<0.05) between the following subgroups of students: female versus male; grade 9 versus grade 12; heterosexual versus gay, lesbian, or bisexual; in-person versus virtual and hybrid; and virtual versus hybrid.

^¶^ Among the 33.7% of students who ever used marijuana, synthetic marijuana, cocaine, or other illegal drugs or misused prescription opioids.

** Pairwise *t*-tests indicate significant differences (p<0.05) between the following subgroups of students: non-Hispanic Asian versus non-Hispanic Black, non-Hispanic multiracial, and non-Hispanic White; non-Hispanic Black versus Hispanic; Hispanic versus non-Hispanic multiracial and non-Hispanic White; grade 9 versus grade 12; and virtual versus hybrid.

^††^ Dashes indicate that results are suppressed because n<30.

## Discussion

This report presents nationally representative data collected beyond the early stages of the COVID-19 pandemic on adolescents’ use of various substances. During spring 2021, during the 30 days before the survey, approximately one in three high school students used any tobacco product, alcohol, or marijuana or engaged in prescription opioid misuse; one in six students used EVPs; one in five drank alcohol; and one in eight used marijuana. In addition, among students who had ever drunk alcohol or used drugs, nearly one in three reported drinking more alcohol or using more drugs during the pandemic. Among students who currently used EVPs or drank alcohol, use on multiple days each month was prevalent, as was binge drinking. These findings are of public health concern because youths’ use of tobacco products in any form is unsafe; EVPs contain nicotine, which is highly addictive, can harm adolescent brain development, and can prime the brain for addiction to other drugs (https://addiction.surgeongeneral.gov/sites/default/files/surgeon-generals-report.pdf). Underage drinking is associated with multiple health risk behaviors, including poor academic performance, injury, violence, and other substance use. Binge drinking is particularly dangerous for adolescents because it can lead to alcohol poisoning (*9*).

The behaviors examined in this report varied by subgroups. Differences by sex, race and ethnicity, grade, and sexual identity were typically consistent with those found in other nationally representative surveys of high school students conducted before the pandemic (*10*,*11*). In addition, this report is the first to provide national substance use data for racial and ethnic groups other than Black, White, and Hispanic. The percentage of AI/AN students using substances was consistently high, indicating a critical need for prevention and intervention strategies to reach these youths. Similarly, the higher prevalence of substance use among gay, lesbian, or bisexual youths than among heterosexual youths underscores the importance of implementing tailored prevention strategies for this population.

Students attending virtual-only schools had a lower prevalence of using each substance examined, which is consistent with the finding that students most commonly obtained EVPs and alcohol from another person. Attending virtual-only schools could lead to lower prevalence of substance use because of fewer social opportunities, less access to substances, and closer parental supervision than attending schools with hybrid or in-person instructional models. However, this finding also might be related to survey setting. Although all students attending virtual-only schools completed the survey at home, students attending hybrid or in-person schools might have completed the survey at school. Students completing surveys at home have demonstrated lower reporting of risk behaviors than students completing surveys at school, possibly because surveys are completed where parents are present, leading to less disclosure of substance use (*12*).

Although approximately one third of students who had ever drunk alcohol or used drugs reported using these substances more during the pandemic, prevalence of current use of all substances examined was lower than in national surveys conducted before the pandemic (*10*,*11*). Although it is unknown whether these decreases are true decreases or a function of differences in survey methods, substance use among youths continues to be a public health concern. Population-based strategies to prevent and reduce youth substance use remain warranted.

Effective prevention of substance use includes strategies that target risk and protective factors at the individual, family, and community levels (https://addiction.surgeongeneral.gov/sites/default/files/surgeon-generals-report.pdf). Population-based strategies have been shown to be effective at reducing youth tobacco product use, including increased pricing of tobacco products, tobacco education campaigns directed at youths, comprehensive smoke-free policies, and restricted youth access to flavored tobacco products (https://www.ncbi.nlm.nih.gov/books/NBK179276/pdf/Bookshelf_NBK179276.pdf). As with tobacco, implementation of alcohol prevention strategies, such as increasing prices, regulating sales, and enforcing laws prohibiting sales to minors, can reduce drinking among both adolescents and adults (https://www.thecommunityguide.org/topic/excessive-alcohol-consumption). Similarly, enhanced use of existing substance use policies, such as prescription drug monitoring programs and safer prescribing practices, can help reduce opioid misuse (*13*). In addition, increasing school connectedness can reduce substance use among students (*14*), although doing so during the pandemic is challenging, which might explain why substance use has increased among certain students. Further, behavioral counseling from health care providers has been demonstrated to be effective in reducing tobacco product use (https://www.uspreventiveservicestaskforce.org/uspstf/recommendation/tobacco-and-nicotine-use-prevention-in-children-and-adolescents-primary-care-interventions); however, youths likely had fewer opportunities for such counseling during the pandemic. Finally, although expanding the delivery of prevention programs that focus on broad-based social-emotional learning and life skills (*15*) and connecting youths to appropriate services (e.g., behavioral health counseling) continue to be critical to preventing youth substance use, youths likely had fewer opportunities to participate in prevention programs and had fewer interactions with health care providers during the pandemic.

## Limitations

General limitations for ABES are available in the overview report of this supplement, including that causality or directionality of observed association cannot be determined (*8*). The findings in this report are subject to at least three specific limitations. First, information about instructional model was provided at the school level rather than the student level. Although students attending virtual-only schools all attended school virtually, and students attending in-person schools all attended school in person, students attending hybrid schools either attended school both in person and virtually or virtually only, making the distinctions between the three groups less certain. Second, other differences between schools using different instructional models, such as location and poverty status, or differences between students in virtual-only versus in-person versus hybrid schools might explain differences in risk behaviors but were not controlled for in analyses. Finally, because alcohol and other drug use are known to increase among adolescents as they age (*10*), the aging of adolescents during the pandemic might account for the reported increase in use of substances among surveyed students. 

## Conclusion

High school students reported substance use during the COVID-19 pandemic, with EVPs, alcohol, and marijuana being the most common; certain respondents who used EVPs and alcohol did so on multiple days during the 30 days before the survey. Further, approximately one in three students who had ever used alcohol and other drugs reported using these substances more during the pandemic. Characterizing youth substance use during the pandemic can inform prevention and intervention strategies and public health messaging during and after the pandemic.
